# A Machine Learning Ensemble Framework for Carbon Price Prediction and Decision Support Under Information Structure Heterogeneity in Regional Carbon Markets in China

**DOI:** 10.3390/e28060656

**Published:** 2026-06-09

**Authors:** Yingyue Xing, Siyuan Zou, Guohua Liu

**Affiliations:** 1 State Key Laboratory of Ocean Engineering, Department of Civil Engineering, Shanghai Jiao Tong University, Shanghai 200240, China; 2 Research Center of Coastal and Urban Geotechnical Engineering, College of Civil Engineering and Architecture, Zhejiang University, Hangzhou 310058, China; 3 Department of Hydraulic Engineering, College of Civil Engineering and Architecture, Zhejiang University, Hangzhou 310058, China; zjuliugh@zju.edu.cn

**Keywords:** carbon price prediction, machine learning, heterogeneous markets, ensemble modeling, LightGBM, decision analytics

## Abstract

Reliable prediction of carbon allowance prices plays a crucial role in emissions trading systems, particularly for market participation, regulatory compliance, and long-term cost planning. In China, regional carbon markets differ markedly in trading activity, price formation mechanisms, and responsiveness to external signals, which limits the effectiveness of conventional single-model forecasting approaches. This study develops a unified machine learning framework designed to accommodate such cross-market heterogeneity. The framework incorporates a diverse set of explanatory variables, including historical price-based indicators, trading volume information, inter-market linkage signals, and macroeconomic factors. Three ensemble-based learning algorithms-XGBoost, LightGBM, and Random Forest—are implemented, and their outputs are further integrated using a weighted aggregation scheme to improve generalization across markets. The empirical evaluation across seven pilot markets shows that, while LightGBM consistently performs well as a standalone model, the proposed ensemble framework achieves superior stability and adaptability under varying market conditions. The forecasting accuracy is high across all cases, with coefficients of determination above 0.74 and reaching values greater than 0.92 in most markets. Further investigation through feature ablation highlights the heterogeneous role of external information, indicating that predictor importance varies significantly between markets and that no universal feature combination yields optimal performance. Leveraging the forecast outputs, the study also demonstrates practical applications in decision support, including timing strategies for allowance sales and dynamic cost assessment in offshore wind engineering scenarios. By systematically evaluating the marginal contribution of different information groups to predictive uncertainty, the framework offers a flexible tool for managing information-structure uncertainty in fragmented carbon markets. The proposed framework offers an integrated solution that connects predictive modeling with operational and engineering decision on processes, providing a flexible tool for managing uncertainty in fragmented carbon markets.

## 1. Introduction

Carbon markets have become an important policy tool for supporting low-carbon transition and reflecting climate-related costs in economic activities. As carbon trading becomes increasingly data-driven, accurate carbon price forecasting is gaining importance for market participants, regulators, and project developers. Carbon price expectations influence not only trading and compliance decisions, but also the estimation of carbon costs in engineering and infrastructure projects [1,2,3,4]. From an information-theoretic perspective, carbon price formation can be viewed as a heterogeneous and policy-sensitive market system in which different markets exhibit marked differences in information processing efficiency, signal-response patterns, and information integration capacity [5]. In highly liquid and continuously traded markets, historical prices alone may already embed most of the relevant information. In contrast, in less mature or sporadically traded markets, trading volume, cross-market price linkages, and macroeconomic signals often play a more important role as additional predictive information [6]. Therefore, cross-market carbon price forecasting is not merely a time-series modeling problem, but rather an information-structure alignment problem—that is, how to identify and integrate the subset of information that is predictively valuable for a given market [7].

China’s carbon market shows strong regional heterogeneity. Before the launch of the national carbon market in 2021, several regional pilot markets had already been established. These regional carbon markets differ in sector coverage, trading activity, market maturity, and price behavior [8,9,10]. Even after the national market was introduced, the regional pilots still provide valuable evidence for understanding carbon price formation under different market conditions. Compared with mature financial markets, these pilot markets often have lower liquidity, stronger policy influence, more irregular trading, and weaker information transmission [11,12]. As a result, carbon price forecasting in China should be understood not only as a technical problem, but also as a problem shaped by market heterogeneity [13].

Previous studies have provided a useful basis for carbon price forecasting. Early research mainly relied on decomposition-based, econometric, and hybrid statistical approaches to address non-stationarity and noise in carbon price series [14,15,16]. Later, machine learning and deep learning methods, including ensemble learning, LSTM-based models, CNN-LSTM, and hybrid forecasting frameworks, were increasingly used to capture nonlinear patterns in carbon markets [17,18,19]. At the same time, external information such as trading volume, energy prices, macroeconomic indicators, news text, and cross-market signals was gradually incorporated into forecasting models [20,21]. This shifted carbon price forecasting from single-series analysis toward multi-source modelling. More recently, greater attention has also been paid to market heterogeneity, interpretability, and application-oriented forecasting [22,23]. Carbon price forecasting involves multiple sources of uncertainty, including market microstructure uncertainty (whether trading is continuous and liquid), external signal uncertainty (whether macroeconomic variables are promptly reflected in prices), and model uncertainty regarding the selection of information combinations (which features are genuinely predictive) [24]. These uncertainties combine differently across markets, making it unlikely that a single information structure performs best in all markets [25,26]. Consequently, a cross-market forecasting framework needs to explicitly assess the marginal contribution of different information groups to predictive uncertainty, which is a core motivation for the feature ablation design in this paper.

Despite these advances, several issues remain insufficiently explored. First, many existing studies focus on model comparison in a single market or on a single dataset [27]. Although this line of research has produced valuable methodological insights, it is less effective for explaining why the value of external information differs across markets. In China’s regional carbon markets, the predictive contribution of trading volume, cross-market linkage, and macroeconomic signals is likely to vary with market maturity, liquidity conditions, and trading continuity [28]. A unified cross-market framework is therefore needed to compare different information structures under heterogeneous market conditions. From a market heterogeneity perspective, China’s regional carbon markets can be viewed as a cluster of heterogeneous regional markets. Each market has its own trading rules, participant structure, and information response function, leading to differentiated predictive values of the same set of external variables across markets [29]. This heterogeneity is not noise but an intrinsic manifestation of market heterogeneity [30]. Therefore, an effective cross-market forecasting framework should not pursue a single universal model but rather provide a method that can adaptively align with market-specific information structures. Following this rationale, this paper uses a unified cross-market modeling framework and a hierarchical feature ablation design to reveal the adaptive boundaries of different information structures in complex market environments [31].

Second, most existing studies focus mainly on forecast accuracy [32]. This focus is both necessary and valuable. At the same time, the practical relevance of carbon price forecasting may extend beyond error reduction alone. Forecast carbon prices can support trading decisions for market participants and may also inform broader decision-making under carbon constraints. They can also be used to estimate future carbon costs in engineering projects that involve embedded emissions or life-cycle carbon accounting. Extending carbon price forecasting to decision-support tasks therefore has clear practical value. Moreover, from an uncertainty quantification perspective, decision-support tasks require not only point forecasts but also an understanding of the reliability and potential variability of predictions—an aspect that remains underexplored in existing the carbon price forecasting literature.

To address these aspects, this study develops a multi-market machine learning framework for carbon price forecasting across China’s regional carbon markets. The purpose is not to propose a new forecasting algorithm, but to compare the performance of different machine learning models across heterogeneous regional markets within a unified framework. The objective of this study is not to conduct an exhaustive benchmark of all recently proposed machine learning algorithms, but to establish a controlled and interpretable cross-market framework for examining how different information structures affect carbon price predictability. Therefore, representative tree-based ensemble models were selected as the modelling basis, rather than introducing a large number of additional model families that may confound market-structure effects with algorithmic complexity. The study also evaluates the added value of trading volume, cross-market features, and macroeconomic variables relative to historical price information. Unlike conventional approaches that assume a universal information structure, our framework explicitly recognizes that the predictive value of external signals is contingent on market-specific conditions—a perspective rooted in the understanding of carbon markets as heterogeneous regional markets. Furthermore, the forecast price paths are extended to two decision-support tasks, namely allowance-selling window identification and dynamic carbon-cost estimation. These tasks are particularly relevant for low-carbon infrastructure projects such as offshore wind farms, where future carbon prices may influence the economic evaluation of alternatives with different carbon footprints. It should be clarified that this study does not aim to provide a formal information-theoretic analysis of carbon-market complexity. The term “information structure” is used in an empirical and feature-based sense, referring to the composition and relative predictive usefulness of price, volume, cross-market, and macroeconomic variables in the forecasting task. Therefore, the analysis focuses on market-dependent feature relevance rather than on formal entropy, mutual information, transfer entropy, or other complexity metrics.

This study makes four main contributions. First, it develops a hierarchical feature ablation framework with five configurations to evaluate the marginal contribution of different feature groups across markets. Second, it provides a unified model comparison across seven regional carbon markets in China, offering a consistent basis for analysing market heterogeneity. Third, it shows that the usefulness of external information is strongly market-dependent, and that no single information structure consistently performs best across all markets. This finding challenges the conventional assumption of a universal feature set and supports the view that adaptive information selection is essential under market heterogeneity. Fourth, it extends carbon price forecasting to two decision-support tasks: allowance-selling window identification and dynamic carbon-cost estimation for offshore wind engineering applications. Within the engineering carbon-cost module, a carbon price threshold is further derived to illustrate potential design-switching conditions under changing carbon market conditions.

The remainder of this paper is organized as follows. Section 2 introduces the data sources, feature construction strategy, and ablation configurations. Section 3 presents the forecasting methodology, model settings, and the extension to decision-support tasks. Section 4 reports the empirical results and discusses market heterogeneity. Section 5 applies the framework to allowance-selling window identification and dynamic carbon-cost estimation. Section 6 concludes the paper.

## 2. Data and Feature Construction

This study examines seven representative regional carbon markets in China: Shanghai, Beijing, Guangdong, Hubei, Tianjin, Fujian, and Shenzhen. These pilot markets were selected because they provide relatively complete trading records and show clear differences in trading continuity, market activity, and price behavior. As shown in Table 1, the seven markets differ in launch year, sample size, and general trading characteristics. These differences provide a suitable basis for testing a unified forecasting framework under heterogeneous market conditions.

For each market, the sample period starts in the launch year and ends in December 2023. Because the launch dates are different, the number of retained observations also varies across markets, ranging from about 1600 to 2500. The target variable is the closing price of carbon emission allowances (CEA). In this study, however, the model predicts the next-day price change rather than the next-day price level. The future price level is then reconstructed from the predicted change series. Price changes were used instead of returns for three reasons. First, several regional carbon markets experienced low-price regimes during part of the sample period, and return-based measures may become unstable or excessively amplified when the price base is low. Second, the downstream decision-support modules, including allowance-selling revenue and carbon-cost estimation, require forecast outputs expressed in CNY/t; price changes can be directly reconstructed into price levels without additional transformation. Third, using absolute price changes provides a consistent and interpretable prediction target for comparing market-specific price paths across heterogeneous regional markets.

**Figure 1:**
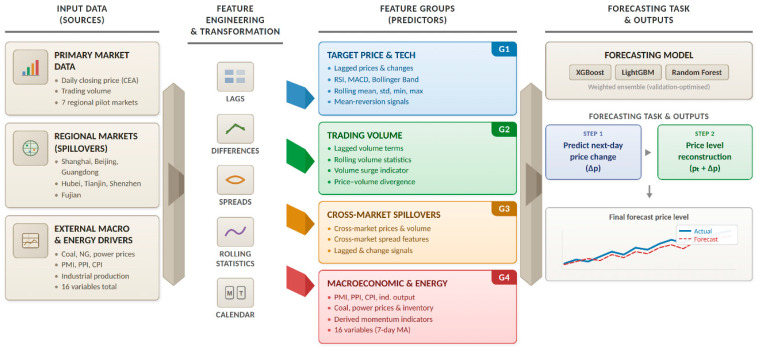
Multi-source feature construction and ensemble forecasting framework for regional carbon markets in China.

As shown in Figure 1, the framework starts from three sources of information: target-market trading data, data from other regional carbon markets, and macroeconomic or energy-related variables. These inputs are then organised into four feature groups and transformed into model-ready predictors. The prediction target is the next-day price change, and the price level is reconstructed from the predicted changes. In this way, Figure 1 summarises the link between raw inputs, feature construction, and the final forecasting task.

### 2.1. Data Preprocessing

The market data were collected from original trading records for the seven regional carbon markets. During preprocessing, the date, price, and, where available, trading-volume fields were identified and standardised across datasets. Price values were converted into numeric form, duplicate daily records were aggregated, and a complete daily calendar was constructed between the first and last available observations. Missing price values were filled by linear interpolation applied to the complete daily calendar. We acknowledge that this procedure may introduce a limited look-ahead risk, because future observed prices can be used when interpolating missing values before lagged predictors are constructed. However, the proportion of missing price observations is low in all seven markets, and the same preprocessing protocol is applied consistently across markets and feature configurations. Therefore, the reported results should be interpreted as a controlled empirical comparison under a consistent preprocessing setting, rather than as a fully leakage-free operational forecasting evaluation. A strictly time-causal imputation strategy, such as forward filling within each training, validation, and test segment, should be adopted in future work. Missing trading-volume values were set to zero to indicate the absence of recorded trading activity on those dates.

Two external data sources were used in the baseline framework. The first was a cross-market dataset built from the other regional carbon markets. For each target market, price series and, where available, trading-volume series from the other pilot markets were aligned by date and merged into the modelling dataset. To avoid target leakage, the target market’s own cross-market columns were removed during training. The second was a daily macro feature table containing 16 macroeconomic and energy-related variables. These variables were merged with the cross-market price and volume data before model fitting.

### 2.2. Feature Groups

Based on the processed market data and external datasets, the predictor set was organised into four feature groups. Depending on the market and the availability of trading-volume data, the final input space contains several dozen variables. As illustrated in Figure 1, these four groups together define the information space used in the forecasting framework.

The first group (G1) contains historical price information and technical indicators derived from the target market itself. These variables include lagged price changes, lagged price levels over multiple short- and medium-term horizons, rolling statistics such as mean, standard deviation, minimum, and maximum, mean-reversion signals, RSI, MACD-related indicators, Bollinger Band position, and calendar variables. Together, these features describe the internal time pattern of the target market and form the core information basis of the model.

The second group (G2) contains trading-volume features. For markets with available volume records, the framework generates lagged volume terms, rolling volume statistics, a volume-surge indicator, and a price–volume divergence signal. These variables are used to capture short-term changes in trading activity and market participation. Their contribution may differ across markets because liquidity conditions and trading continuity vary substantially among China’s regional carbon markets.

The third group (G3) contains cross-market linkage features. To reflect interactions across regional markets, the framework incorporates both cross-market prices and cross-market trading volumes from the other regional markets. These external series are aligned by date and then transformed into lagged and change-based signals. In addition, spread features are constructed for cross-market price series as the difference between each external market price and the target market price. This allows the model to capture both short-term co-movement and relative price positions across regional carbon markets.

The fourth group (G4) contains macroeconomic variables. In the baseline setting, the macro feature table includes 16 variables. As shown in Table 2, these variables fall into three categories: economic activity, energy and power, and derived or momentum indicators. These variables provide additional information on broader economic conditions and energy-market movements that may affect short-term carbon price dynamics.

A key principle of the feature design is that external information enters the model mainly in transformed form rather than as raw series alone. This helps reduce scale differences across heterogeneous data sources and improves consistency between the predictors and the next-day price-change target. As indicated in Figure 1, lag terms, first differences, spread signals, rolling statistics, and calendar variables are used to convert raw inputs into model-ready features.

The same macroeconomic and energy-variable pool was used for all seven markets to ensure cross-market comparability. Since the objective of this study is to examine how the same information structure performs under heterogeneous market conditions, adopting market-specific covariate sets at the preprocessing stage would introduce an additional source of subjective selection bias. Instead, market heterogeneity is evaluated through the ablation design in Section 2.3, where the marginal contribution of volume, cross-market, and macroeconomic information is compared across markets. Therefore, the unified 16-variable macroeconomic pool should be interpreted as a controlled common information set rather than as a claim that all variables are equally relevant to every market.

### 2.3. Ablation Configuration

To assess the contribution of each feature group in a systematic way, this study adopts a hierarchical ablation design with five configurations, as shown in Table 3: M1 (Full Model, including all four feature groups), M2 (without volume features), M3 (without cross-market features), M4 (without macroeconomic variables), and M5 (Price-Only, retaining only the target market’s own historical price information and technical indicators). The baseline full-feature setting is aligned with M1 so that the ablation results directly reflect the marginal effect of removing a given feature group, without being affected by additional differences in model setting. As shown in Table 3, G1 is retained in all five configurations because it represents the core internal information of the target market. By contrast, G2, G3, and G4 are removed one by one or jointly excluded to test the added value of trading-volume information, cross-market linkage, and macroeconomic factors. This design provides a clear basis for comparing the role of different information groups across markets under a consistent modelling framework. These five configurations form a hierarchical information removal sequence, allowing us to quantify the marginal contribution of each feature group to predictive uncertainty. From an information-theoretic perspective, this design is equivalent to evaluating the “empirical predictive contribution” of different information sources under heterogeneous market conditions.

## 3. Methodology

### 3.1. Forecasting Models and Ensemble Strategy

This study adopts a unified forecasting framework built on three tree-based predictive models: XGBoost, LightGBM, and Random Forest. Tree-based models are chosen because they naturally handle mixed-type predictors, are robust to feature-scale differences across heterogeneous data sources, and are well suited to medium-sized tabular datasets. In addition, all three models provide feature importance scores, which can be used for post hoc inspection of model behavior across different regional markets.

XGBoost, LightGBM, and Random Forest were selected because they represent two widely used tree-based ensemble-learning mechanisms: boosting and bagging. XGBoost and LightGBM provide sequential boosting structures that are effective for capturing nonlinear relationships, whereas Random Forest provides a bagging-based benchmark with different variance-reduction behaviour. This combination offers model diversity while keeping the comparison transparent and consistent across seven markets and five feature configurations [33].

XGBoost minimises a regularised objective function that combines a differentiable loss term with explicit L1 and L2 penalties on tree weights, which helps control overfitting in a high-dimensional feature space. LightGBM uses a leaf-wise tree growth strategy together with histogram-based binning, which improves computational efficiency when the feature set becomes large after cross-market and macroeconomic variables are included. Random Forest builds an ensemble of independently grown trees through bootstrap aggregation, and its structural difference from the two boosting models provides useful model diversity in the final ensemble.

The main hyperparameter settings of the three base learners are summarised in Table 4. To ensure consistency in cross-market comparison, the same settings are used across all seven regional carbon markets. These settings were determined through a coarse validation-set grid search on two representative markets, Shanghai and Hubei, which differ in trading activity and market characteristics. The search focused on the main complexity-control parameters of the tree-based models, including tree depth, number of leaves, minimum child samples, learning rate, number of boosting rounds, and regularisation strength. The selected settings were those that provided a reasonable balance between model flexibility and overfitting control under different liquidity conditions. Market-specific hyperparameter tuning was not performed, because the purpose of this study is to maintain a controlled basis for cross-market and cross-feature-configuration comparison. Although market-specific tuning may improve local predictive accuracy, it would introduce an additional source of variation and make it more difficult to attribute performance differences to feature-group effects. The potential benefit of market-specific tuning is further discussed in the limitations.

The three base models are further combined through a weighted ensemble. Rather than assigning fixed weights in advance, the framework determines the optimal convex combination on the validation set through grid search. The weight combination is selected by minimising the mean squared error on the validation set. In this sense, the proposed ensemble is not a simple arithmetic average, but an optimization-based weighted ensemble in which model weights are selected by minimizing validation-set MSE. This lightweight optimization strategy is intended to improve the adaptability of the ensemble while preserving the comparability of the cross-market experiment. This ensemble design does not assume that the ensemble always outperforms every individual model. Instead, it is intended to provide a more balanced benchmark across heterogeneous markets.

### 3.2. Training, Validation, and Evaluation Protocol

The observations are split chronologically into training, validation, and test sets at a fixed ratio of 75:12:13. This temporal split avoids look-ahead bias and ensures that future information does not enter model fitting. The 75:12:13 ratio was chosen to balance three requirements: retaining sufficient historical observations for model training, reserving an independent validation period for early stopping and ensemble-weight optimization, and keeping a temporally separated test period for final out-of-sample evaluation. Because the seven markets have different launch dates and sample sizes, a fixed proportional split also ensures that the training, validation, and test periods are defined consistently across markets. The validation set serves two purposes: early stopping for the boosting models and ensemble-weight selection. The test set is reserved exclusively for final out-of-sample evaluation.

In this framework, the prediction target is the next-day price change rather than the next-day price level itself. After the one-step-ahead change is predicted, the corresponding price level is reconstructed by adding the predicted change to the last observed price. All reported evaluation metrics are computed on reconstructed price levels rather than on predicted price changes. This setting is used to improve the handling of non-stationarity in price levels while preserving the practical interpretability of the forecasting results (see Table 5 for a summary of the training and evaluation protocol).

For multi-step future forecasting in the decision-support application, the framework uses recursive updating, in which each predicted price is appended to the historical series before the next step is computed. Forecast accuracy is expected to decline as the horizon extends due to error accumulation, and long-horizon results should therefore be interpreted with caution.

One additional feature engineering detail should be noted. Several macroeconomic variables are published at monthly frequency. When these series are converted into daily differenced signals, artificial spikes may appear at the beginning of each month. To reduce this problem, the framework applies a seven-day moving average to the differenced macroeconomic series before they are used in model training.

Forecast performance is evaluated using four metrics: RMSE, MAE, MAPE, and R^2^. RMSE is treated as the primary metric because it penalises larger errors more heavily and is more sensitive to substantial deviations in reconstructed price levels. MAE provides an absolute-error complement, MAPE offers a scale-relative perspective, and R^2^ measures the proportion of price variation explained by the model. Because MAPE can be amplified in markets with relatively low price levels, it is treated as a secondary rather than primary criterion in those cases. The benchmark set in this study is limited to representative tree-based machine learning models and their weighted ensemble. Classical econometric benchmarks, such as ARIMA, GARCH, and random-walk-type models, are not included in the present version. Therefore, the empirical results should be interpreted as a controlled comparison within a machine-learning framework rather than as an exhaustive benchmark against the full carbon-price forecasting literature.

### 3.3. Decision-Support Methodology

Beyond one-step forecasting, the framework is extended to two decision-support tasks. These extensions are intended as application-oriented decision aids rather than fully validated decision-support demonstrations. First, the predicted future price path is used to identify favourable carbon allowance selling windows. Under a user-specified allowance quantity, forecast prices are converted into expected revenues, and both the single forecast-based best selling day and a set of high-return windows defined by a quantile threshold are identified over the forecast horizon.

Second, forecast prices are coupled with life-cycle carbon footprint estimates from engineering scenarios to quantify dynamic carbon cost under different design choices and to derive a threshold-based threshold-based screening indicator for engineering design comparison. In the offshore wind energy context, this extension makes it possible to compare alternative scour protection strategies under changing carbon-price expectations. In this way, the role of the forecasting framework is extended from market prediction to engineering-oriented decision support.

For multi-step forecasting, the empirical forecast range is constructed as the point forecast plus or minus 1.96 times the standard deviation of out-of-sample residuals. Under the assumption that forecast errors accumulate approximately independently across steps, the residual standard deviation is scaled by the square root of the forecast step number. This range should be interpreted as an empirical uncertainty band rather than a formal statistical confidence interval.

The framework described above is applied to each regional market under the five feature configurations defined in Section 2.3, and the resulting performance is reported in Section 4.

The engineering case used in the carbon-cost extension is based on a refined O&M-LCA model developed by the authors for a 202 MW offshore wind farm in southeastern China. The model compares two representative scour protection strategies for monopile and high-pile cap foundations: S1, rock dumping scour protection, and S2, cement-stabilised soil scour protection. S1 involves periodic quarrying, land–sea transport, and offshore placement of crushed rock around foundations, with maintenance frequency depending on hydrodynamic conditions. S2 replaces rock with in situ seabed stabilisation using cementitious binders, with material demand depending on geological conditions and stabilisation efficiency. The LCA model was developed following ISO 14040/14044 [34,35] and the ReCiPe 2016 Midpoint method, with the functional unit defined as 1 MWh of net electricity delivered over a 25-year design life.

The LCA results show a clear environmental trade-off between the two strategies. S1 causes only a modest increase in global warming potential (GWP) relative to the baseline O&M scenario (from 4.36 to 4.55 kg CO_2_-eq/MWh under the medium-frequency scenario), but substantially increases air-pollution- and mineral-resource-related burdens due to large-scale quarrying and long-distance transport. S2 reduces mineral resource scarcity by 98% compared with S1, but raises GWP to 9.94 kg CO_2_-eq/MWh due to cement production and offshore treatment. Three scenarios were defined for each strategy to reflect site-condition uncertainty: for S1, low-frequency (2 interventions/25 yr), medium-frequency (5 interventions/25 yr, baseline), and high-frequency (8 interventions/25 yr) maintenance; for S2, fast-stabilisation, medium-stabilisation (baseline), and slow-stabilisation conditions. Table 6A summarises the scenario-level life-cycle carbon emissions used in the decision-support extension.

For the engineering cost comparison, the direct cost difference between S1 and S2 is treated as a user-defined parameter rather than a single fixed value, because it depends on site-specific factors such as material prices, transport distance, vessel availability, and construction conditions. The carbon price threshold is calculated as follows:

(1) $$P^{*}=\frac{{\Delta C}_{direct}}{∆E}$$

where *P*^∗^ denotes the carbon price threshold, ΔC_direct_ is the direct cost premium of S1 over S2, and ΔE is the emission difference between S2 and S1. In the baseline comparison (S1 mid vs. S2 mid), ΔC_direct_ is set to 15 million CNY, representing the direct cost premium of S1 over S2. 

The sensitivity of the carbon price threshold P^*^ to ΔC_direct_ is further examined in Section 5.2.3. The emission difference between S1 mid and S2 mid is 68,593 t CO_2_-eq, which yields the baseline threshold P* = 218.7 CNY/t as derived in Section 5.2.2.

## 4. Results and Discussion

### 4.1. Baseline Model Performance Under the Full-Feature Configuration

This section presents the empirical results of the proposed multi-market forecasting framework. Under the full-feature configuration (M1) defined in Section 2, all baseline models were trained on the same information space, including historical price indicators, trading volume, cross-market linkage features, and macroeconomic variables. This unified setting provides a controlled basis for comparing the three individual models and the weighted ensemble across the seven regional carbon markets.

**Figure 2:**
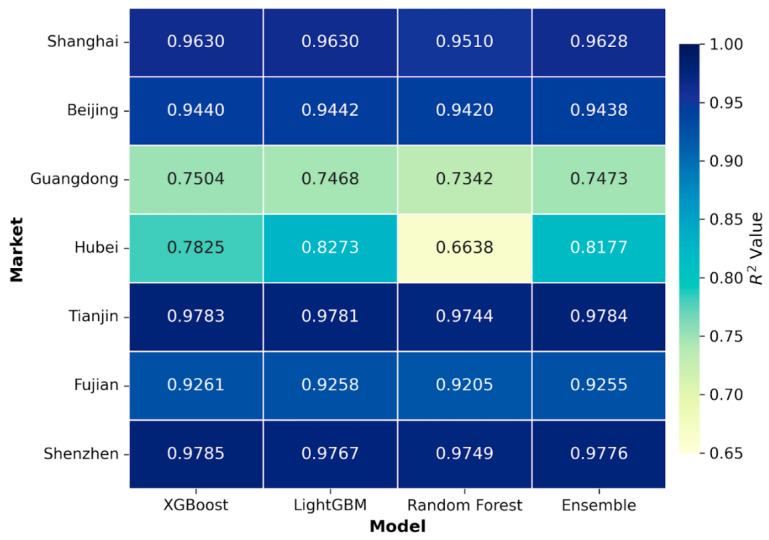
Heatmap of model goodness-of-fit (R^2^) under the baseline full-feature configuration.

As shown in Table 7 and Figure 2, the weighted ensemble achieves an average R^2^ of 0.908 across the seven markets, accompanied by an average RMSE of 2.638 CNY/t, an average MAE of 1.567 CNY/t, and an average MAPE of 4.69%. These results indicate that the full-feature framework reconstructs carbon price paths with high accuracy, even under the substantial heterogeneity observed across regional market conditions.

A closer comparison of the individual models shows that no single algorithm dominates in all seven markets. Among the three base learners, LightGBM delivers the strongest average performance, with the lowest mean RMSE (2.638) and the highest mean R^2^ (0.909), followed closely by XGBoost. Random Forest consistently performs worse than the two boosting models. A likely reason is that its bagging structure is less effective than sequential boosting in capturing the short-term nonlinear dependencies in carbon price series. The weighted ensemble does not outperform LightGBM in every market. However, it shows a more balanced performance profile across the full set of heterogeneous markets. For this reason, the ensemble is retained as the unified benchmark in the subsequent ablation analysis, rather than being treated as the universally best model.

Substantial cross-market heterogeneity is evident in the baseline results. The ensemble reaches R^2^ values above 0.96 in Tianjin, Shenzhen, and Shanghai, indicating near-complete price reconstruction in these markets. Beijing also performs well, with an R^2^ of about 0.94.

By contrast, Guangdong (R^2^ ≈ 0.75) and Hubei (R^2^ ≈ 0.82) are notably harder to forecast. This contrast likely reflects differences in market microstructure and price dynamics. To support this interpretation, Table 6B reports full-sample descriptive statistics of daily CEA prices and trading activity for Guangdong and Hubei. Guangdong is characterized by stronger price volatility: its price coefficient of variation reaches 69.0%, compared with 37.7% for Hubei; the standard deviation of daily log-returns is 7.73%, compared with 4.10% for Hubei; and 23.2% of its trading days record an absolute daily return above 5%, compared with 13.7% for Hubei. In addition, Guangdong shows a wider price range and larger single-day price jumps, with the maximum single-day price change reaching 49.75 CNY/t. These features indicate that Guangdong’s price path contains stronger dispersion and more frequent abrupt movements, which is consistent with its relatively larger reconstruction error and lower R^2^ in the baseline results.

Hubei, by contrast, does not show the same level of continuous price volatility. The phrase “more irregular trading patterns” refers mainly to stable price levels combined with uneven and burst-like trading activity. Although Hubei has a comparable average number of trading days per year, its trading-volume coefficient of variation reaches 255%, and the busiest 5% of trading days account for about 37% of total turnover. This suggests that Hubei alternates between relatively quiet periods and short episodes of concentrated trading, rather than exhibiting a smooth and continuous trading process. Therefore, the remaining forecasting difficulty in Hubei appears to be more closely associated with intermittent trading activity and heavy-tailed price changes than with persistent high volatility as observed in Guangdong. These market-microstructure features create additional forecasting difficulty that the model cannot fully resolve.

### 4.2. Feature Ablation Analysis and Market-Dependent Information Value

**Figure 3:**
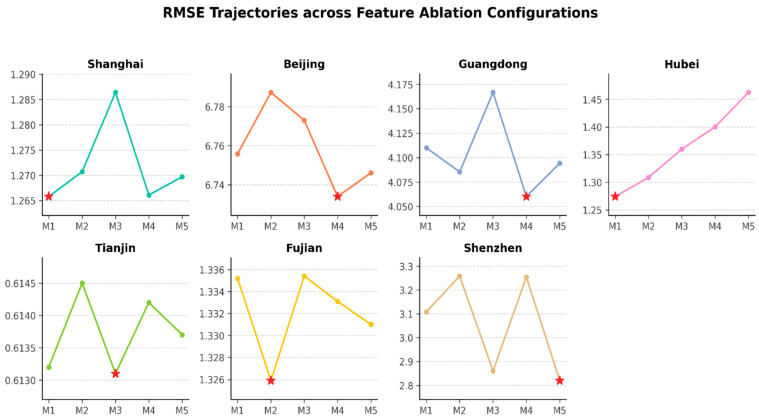
Predictive error (RMSE) under different feature configurations, with the optimal feature set for each market indicated by a red star.

To examine the contribution of each feature group, the five ablation configurations defined in Section 2.3 were evaluated across all seven markets. The weighted ensemble was used as the unified benchmark in this analysis, consistent with its role in Section 4.1. The ablation results suggest that the predictive value of external information is market-dependent, although the magnitude of the differences varies across markets. No single feature configuration achieves the lowest RMSE in all markets. Table 8 and Table 9 report the detailed and summary results, and Figure 3 shows the RMSE trajectories across configurations. It should be noted that some performance differences across feature configurations are numerically small. Therefore, the ablation results should be interpreted as indicative evidence of market-dependent information suitability rather than as statistically definitive proof of distinct information structures.

Several patterns can be identified. First, the full model (M1) performs best in Shanghai and Hubei. In Shanghai, however, the advantage is only marginal, indicating that performance is only weakly affected by feature selection. In Hubei, by contrast, M1 shows a clearer advantage, suggesting that external information provides meaningful additional predictive value.

Second, reduced configurations perform better in several markets. Beijing and Guangdong achieve the lowest RMSE under M4, indicating that macroeconomic variables do not improve short-term forecasting in these two markets and may instead introduce noise. Tianjin performs best under M3, although the differences across configurations are very small. Fujian performs best under M2, suggesting that volume-related signals are less informative in a market with discontinuous trading activity.

Third, Shenzhen shows a distinct pattern. The price-only model (M5) gives the lowest RMSE, while M3 also outperforms M1. This suggests that Shenzhen’s local price dynamics differ from those of the other markets, so additional external inputs, especially cross-market signals, may weaken rather than improve prediction.

Overall, the ablation results show that there is no universally optimal information structure across China’s regional carbon markets. The usefulness of volume, cross-market, and macroeconomic features depends on the characteristics of the target market. These findings support the main argument of this study: carbon price forecasting in China is shaped not only by model choice, but also by market structure and information suitability.

These results reveal a regularity at the level of market heterogeneity: a market’s information-processing structure is closely related to its trading activity and institutional maturity. In highly liquid and continuously traded markets (e.g., Shanghai), all information groups are effectively absorbed, making the full model (M1) optimal. In less mature or sporadically traded markets (e.g., Shenzhen, Tianjin), external signals not only fail to help but may introduce “informational noise” that mismatches the local price formation mechanism. This suggests that predictive uncertainty in carbon markets arises not only from data noise but also from mismatches between information structure and market microstructure. A unified forecasting framework should not pursue a universal feature combination but rather provide the ability to identify such mismatches and adaptively select the relevant information subset.

Since formal forecast-comparison tests, such as Diebold–Mariano tests or bootstrap confidence intervals, are not implemented in this study, conclusions based on small RMSE or R^2^ differences should be interpreted cautiously. The ablation analysis is therefore intended to provide an exploratory comparison of feature-group relevance under a unified modelling protocol, rather than a statistical test of dominance among feature configurations.

### 4.3. Metric Interpretation and Cross-Market Synthesis

Among the four evaluation metrics, RMSE and R^2^ are given the greatest weight. RMSE directly measures reconstruction error in CNY/t and is therefore the most relevant metric for the decision-support tasks developed in Section 5. R^2^ complements RMSE by showing how much price variation is captured by the model. MAE serves as a useful absolute-error supplement. MAPE is reported only as a supplementary metric because percentage-based errors can become unstable when price levels are low or when markets experience prolonged low-price regimes. Therefore, the interpretation of model performance in this study relies primarily on RMSE and R^2^ rather than on MAPE alone. This issue is most visible in Shenzhen. Under the baseline configuration, Shenzhen shows a high R^2^ but also a relatively high MAPE. These results are not contradictory. The model captures the overall price trajectory well, but percentage errors are amplified because Shenzhen had a low-price regime in the early sample period. Similar caution may also apply to other markets when price levels are low. Future work should complement MAPE with more robust alternatives, such as sMAPE, MASE, RMSLE, or QLIKE-type metrics, especially for markets with prolonged low-price regimes.

This issue is most visible in Shenzhen. Under the baseline configuration, Shenzhen shows a high R^2^ but also a relatively high MAPE. These results are not contradictory. The model captures the overall price trajectory well, but percentage errors are amplified because Shenzhen had a low-price regime in the early sample period. Similar caution may also apply to other markets when price levels are low. Future work should complement MAPE with more robust alternatives, such as sMAPE, MASE, RMSLE, or QLIKE-type metrics, especially for markets with prolonged low-price regimes.

This issue is most visible in Shenzhen. Under the baseline configuration, Shenzhen shows a high R^2^ but also a relatively high MAPE. These results are not contradictory. The model captures the overall price trajectory well, but percentage errors are amplified because Shenzhen had a low-price regime in the early sample period. The ablation results further show that Shenzhen performs best under the price-only configuration, indicating that the high MAPE should not be interpreted as weak overall model quality.

The cross-market comparison also shows that model evaluation cannot be reduced to a single best specification. Beijing and Guangdong perform best without macroeconomic variables, Shanghai and Hubei perform best under the full model, Tianjin performs best without cross-market features, Fujian performs best without volume features, and Shenzhen performs best under a price-only setting. These results confirm that the predictive value of external information depends on market context. In practice, forecasting performance depends not only on algorithm design, but also on whether the information structure matches the characteristics of the target market.

## 5. Decision-Support Extension

Section 4 showed that the proposed framework can reconstruct carbon price paths with relatively strong accuracy in most regional markets within the tree-based benchmark considered in this study, although predictive performance remains market-specific. Based on these results, this section extends the forecasting framework to two practical applications: allowance-selling window identification in Section 5.1 and dynamic carbon-cost estimation for engineering applications in Section 5.2. It should be emphasized that the decision-support extension is intended as an illustrative application rather than a fully validated operational optimization framework. The main methodological focus of this paper remains the cross-market carbon price forecasting framework and the evaluation of market-dependent feature relevance. The allowance-selling and engineering carbon-cost modules are included to demonstrate how forecast price paths can be translated into practical decision signals when allowance quantities or life-cycle emission estimates are already available. Therefore, the offshore wind LCA case serves as an application interface between carbon-price forecasting and engineering decision analysis, rather than as an independent LCA contribution within this paper. The second application further introduces a carbon price threshold, P*, to illustrate potential design-switching conditions under changing market conditions. Since both applications rely on reconstructed future price levels, the predictive accuracy reported in Section 4, especially RMSE, directly affects the reliability of the downstream outputs.

### 5.1. Allowance-Selling Window Identification

**Figure 4:**
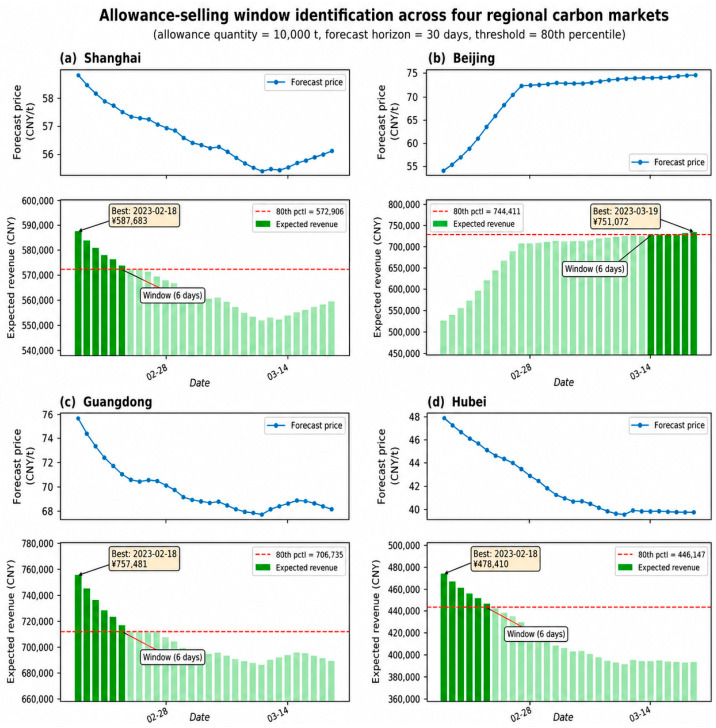
Allowance-selling window identification across four representative regional carbon markets under a 30-day forecast horizon and an allowance quantity of 10,000 t. (**a**) shows the forecast carbon price path for Shanghai, (**b**) shows the corresponding expected revenue sequence for Beijing, (**c**) shows the forecast carbon price path for Guangdong, (**d**) shows the corresponding expected revenue sequence for Hubei. The dashed line denotes the 80th-percentile threshold, and the light green bars indicate the expected revenue.

For each market, the forecast carbon price path over a selected horizon is converted into an expected revenue sequence for a user-defined allowance quantity, following the procedure described in Section 3.3. The framework then provides two outputs: the forecast-based bestselling day, defined as the date with the highest expected revenue within the forecast horizon, and the forecast-based high-return candidate window, defined as the set of dates on which expected revenue exceeds a selected quantile threshold of the forecast revenue distribution. Unless otherwise stated, the examples in this section use the market-specific best-performing configuration identified in Section 4. In the baseline setting, the allowance quantity is fixed at 10,000 t, the forecast horizon is 30 days, and the 80th percentile is used as the default threshold. This choice is intended to balance selectivity and execution flexibility. A lower threshold, such as the 70th percentile, would generate a broader candidate window but weaken the screening effect, whereas a higher threshold, such as the 90th percentile, would identify only a very small number of top-return dates and may be too restrictive in practice. The 80th percentile is therefore used here as a practical default rather than a universal optimum. Figure 4 presents the allowance-selling results for four representative regional carbon markets: Shanghai, Beijing, Guangdong, and Hubei. In each case, panel (a) shows the forecast price path, and panel (b) shows the corresponding expected revenue sequence together with the threshold and the identified high-return window.

A clear cross-market contrast can be observed. In Shanghai, Guangdong, and Hubei, the forecast price path shows an overall downward tendency over the 30-day horizon. As a result, the forecast-based bestselling day appears near the beginning of the forecast window, and the high-return window is concentrated in the early part of the period. By contrast, Beijing shows a rising forecast path over most of the horizon, so the forecast-based bestselling day shifts to the end of the forecast period and the identified window are concentrated in the later stage. Under the baseline threshold, all four cases produce a six-day high-return window, indicating that the 80th-percentile rule provides a practical balance between selectivity and execution flexibility. These results show that the module does not impose a fixed early-selling or late-selling rule, but converts each market-specific forecast trajectory into an explicit timing recommendation.

Overall, this application illustrates how carbon price forecasting can be extended from a predictive task to a timing-oriented decision tool. Its purpose is not to guarantee realised trading gains, since actual outcomes still depend on future market movements and execution conditions. Rather, its value lies in providing a transparent basis for identifying favourable selling periods under a user-defined forecast horizon and allowance quantity.

### 5.2. Dynamic Carbon-Cost Evaluation for Engineering Applications

#### 5.2.1. Dynamic Carbon-Cost Trajectories Under Forecast Price Paths

Beyond trading timing, forecast carbon prices can also be used to estimate future carbon costs in engineering applications. In this study, the approach is demonstrated through the comparison of two offshore wind scour protection strategies introduced in the earlier LCA analysis, namely S1 and S2. Let denote the scenario-level life-cycle carbon emission of option, and let denote the forecast carbon price on day. Specifically, the scenario-level life-cycle carbon emissions of each option are combined with the forecast carbon price to estimate its corresponding carbon cost under future market conditions.

This cost measure should not be interpreted as a carbon tax or an actual payment obligation. In this short-horizon application, is treated as a scenario-level fixed carbon quantity for comparative pricing rather than as a temporally distributed emission flow. The purpose is not to perform full temporal carbon accounting, but to examine how short-term changes in carbon price expectations affect the relative carbon-cost ranking of engineering alternatives.

The difference in carbon cost between two competing options can therefore be evaluated by comparing their life-cycle carbon emissions under the same forecast carbon price path. Because S2 has the higher life-cycle carbon burden in the baseline comparison, rising carbon prices increase the relative carbon-cost disadvantage of S2 over time, while lower carbon prices reduce the magnitude of this difference.

**Figure 5:**
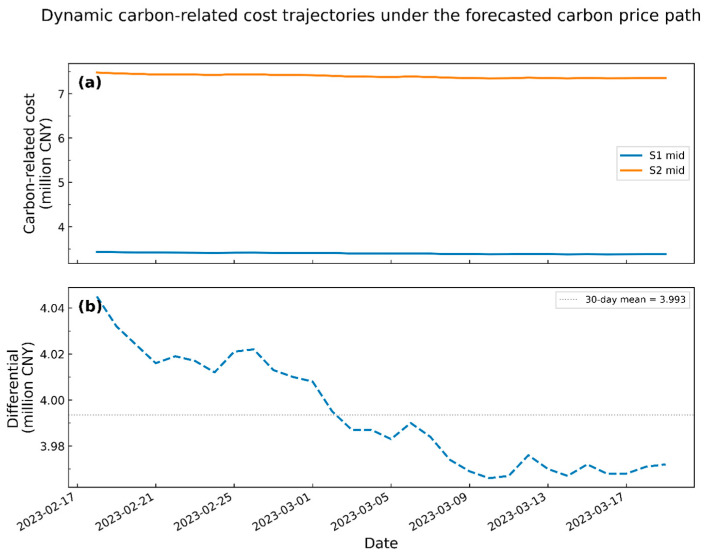
Dynamic carbon cost trajectories under the forecast carbon price path for the representative baseline comparison. Panel (**a**) shows the carbon costs of S1 mid and S2 mid. Panel (**b**) shows the corresponding cost differential over the 30-day forecast horizon.

Figure 5 illustrates the dynamic carbon cost trajectories of the two strategies under the forecast carbon price path. The comparison shows that the cost gap attributable to carbon emissions changes directly with the predicted market price level. Even within a relatively short forecast horizon, the framework can translate changes in carbon price expectations into changes in comparative engineering cost signals. This provides a more decision-relevant interpretation than a single static carbon price assumption.

#### 5.2.2. Carbon Price Threshold for Design Switching

A more directly illustrative decision signal is the carbon price threshold at which the total-cost ranking of S1 and S2 reverses. Let denote the direct cost premium of S1 over S2, and let denote the emission reduction achieved when switching from S2 to S1. The threshold carbon price can then be obtained by dividing the direct cost premium by the corresponding emission reduction. This threshold is meaningful only when S1 has a higher direct cost but lower life-cycle emissions than S2, because in this case there is a clear trade-off between economic cost and carbon performance. If S1 is both cheaper and cleaner, it dominates unconditionally and no switching threshold is needed.

The interpretation is straightforward. It represents the carbon price at which the carbon-cost advantage of S1 exactly offsets its additional direct cost. When the forecast carbon price exceeds, S1 becomes preferable on a total-cost basis. When the forecast carbon price remains below, S2 retains its economic advantage. For the baseline comparison between S1 mid and S2 mid, the threshold is 218.7 CNY/t. This value serves as the benchmark for the forecast-to-threshold comparison in Section 5.2.4.

**Figure 6:**
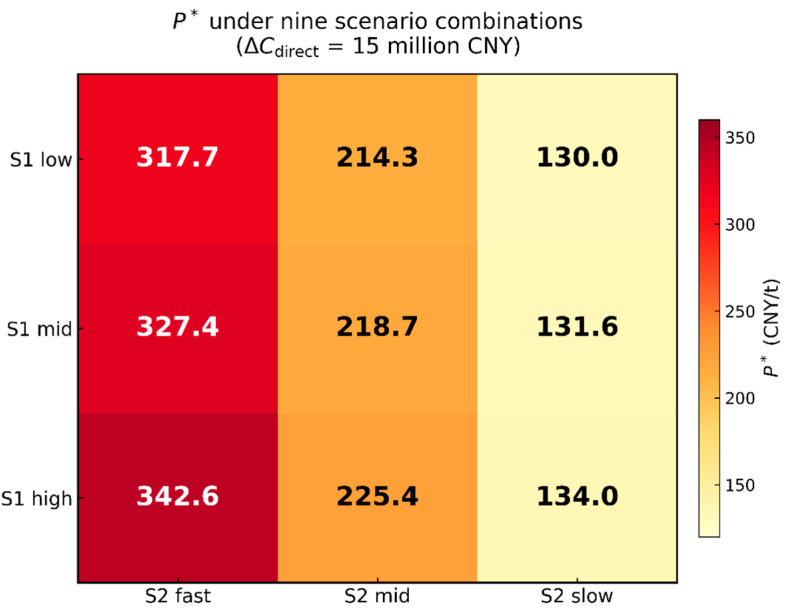
Carbon price threshold under nine scenario combinations at million CNY. Rows represent the S1 low, mid, and high cases. Columns represent the S2 fast, mid, and slow cases. P* denotes the carbon price threshold at which the total-cost preference between the two engineering options may change.

Figure 6 reports under all nine scenario combinations at 15 million CNY. Two patterns can be observed. First, ranges from 130.0 to 342.6 CNY/t, which confirms that the switching threshold is not a fixed engineering constant but a scenario-dependent quantity shaped jointly by the intervention frequency assumed for S1 and the stabilisation condition assumed for S2. Second, decreases clearly from the S2 fast column to the S2 slow column, which indicates high sensitivity to the emission performance of the higher-emission route. By comparison, variation across the S1 rows is more moderate. These results show that the threshold framework can be naturally extended to a scenario matrix and can identify the carbon-price conditions under which different pairwise comparisons become decision-relevant.

#### 5.2.3. Sensitivity and Uncertainty Interpretation

The threshold result is affected by uncertainty in both engineering and life-cycle parameters. In practice, the direct cost premium may vary with material prices, transport distance, vessel availability, construction methods, and site-specific conditions. Similarly, the estimated emission reduction depends on LCA system boundaries, emission-factor selection, and scenario assumptions regarding material use, transportation, and construction activities. Therefore, the switching threshold should not be treated as a single fixed engineering constant. Instead, it should be understood as a scenario-dependent result shaped by both cost-side and emission-side assumptions.

Equation (1) reveals two sensitivities directly. First, increases linearly with. A larger direct cost premium requires a higher carbon price to justify the lower-emission option. Second, decreases as increases. A larger emission gap means that even a moderate carbon price can alter the economic ranking, whereas a smaller gap requires a much higher price to produce the same effect. These two relationships explain why the threshold varies across the nine scenario combinations introduced in Section 5.2.2.

**Figure 7:**
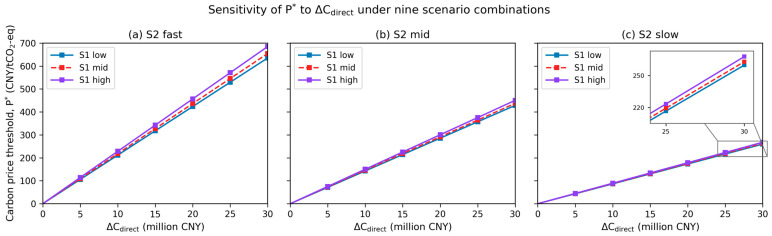
Sensitivity of the carbon price threshold to direct cost difference under nine scenario combinations. P* denotes the carbon price threshold at which the total-cost preference between the two engineering options may change.

Figure 7 shows the sensitivity of the switching threshold to the direct cost premium under the nine scenario combinations. All curves increase linearly, which is consistent with Equation (1), but their slopes differ markedly. Combinations with a smaller emission gap produce steeper lines and higher thresholds, whereas those with a larger produce flatter lines and lower thresholds. Taken together, the nine curves define a scenario-dependent threshold family rather than a single fixed value. This result is important because it shows that the economic relevance of carbon pricing depends not only on the forecasted market price, but also on the underlying engineering and life-cycle assumptions used in the comparison.

Forecast uncertainty should be considered together with engineering uncertainty. In this study, the forecast band is constructed using the empirical approach described in Section 3.3, namely the point forecast plus or minus 1.96 times the out-of-sample residual standard deviation, scaled by the square root of the forecast step. Under this setting, the decision implication does not depend only on whether the point forecast crosses, but also on how the full forecast band is positioned relative to the threshold region.

Table 9 summarises three possible forecast-to-threshold relations and their engineering implications. When the forecast band lies clearly below, the higher-emission option is likely to retain its total-cost advantage, and the direct-cost advantage remains dominant. When the forecast band is close to or overlaps, the ranking becomes sensitive to forecasting and scenario assumptions, so the engineering preference should be treated as conditional. When the forecast band lies clearly above, the lower-emission option may become preferable in total-cost terms, indicating that carbon pricing has become strong enough to reshape the design preference. In this way, the threshold framework provides a simple but interpretable rule for linking forecast uncertainty to engineering comparison. By incorporating both forecasting uncertainty and engineering parameter uncertainty into a unified framework, the decision implication of the carbon price threshold is no longer a simple “whether the price exceeds a certain value,” but rather a robustness judgment under multiple uncertainties. This approach reflects the intrinsic link between information integration and decision-making in complex systems.

#### 5.2.4. Forecast-to-Threshold Comparison

**Figure 8:**
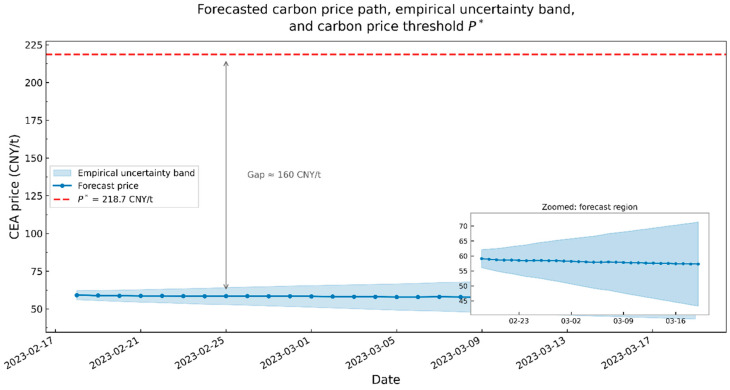
Forecast carbon price path, empirical uncertainty band, and representative carbon price threshold for the baseline engineering comparison. P* denotes the carbon price threshold at which the total-cost preference between the two engineering options may change.

Figure 8 overlays the forecast carbon price path and its empirical uncertainty band with the baseline threshold CNY/t. The contrast is clear. The entire forecast path, including the upper bound of the uncertainty band, remains far below throughout the 30-day horizon. Under the current price expectations, the forecast carbon price is therefore not high enough to offset the direct cost premium of S1 over the analysed horizon. In other words, carbon pricing does not reverse the current total-cost ranking in the baseline comparison.

At the same time, the threshold framework identifies the carbon price level at which S1 would begin to gain an economic advantage. This question becomes increasingly relevant under longer planning horizons or under a future market environment with tighter emission constraints and higher carbon prices. From this perspective, the value of the framework lies not only in judging whether carbon pricing matters today, but also in identifying when it may start to matter in future design choices.

#### 5.2.5. Broader Relevance

Although this section demonstrates the method through offshore wind scour protection, the threshold logic is not limited to this case. It can also be applied to a broader range of engineering decisions in which competing options differ in both direct cost and life-cycle carbon emissions. Examples include concrete mix design with different clinker substitution levels, material substitution in structural components, and comparisons between conventional and lower-carbon manufacturing routes. In each case, the threshold formula remains unchanged, while the input values are redefined according to the specific engineering context.

More broadly, linking carbon price forecasting with dynamic carbon-cost estimation provides a methodological bridge between market prediction and life-cycle decision analysis. By embedding dynamic price expectations into design comparison, the framework moves beyond the common practice of using a fixed carbon price assumption in LCA-informed engineering studies. It therefore offers a more flexible basis for low-carbon infrastructure planning and engineering decision support.

## 6. Conclusions

This study presents a multi-market machine learning framework for carbon allowance price forecasting across seven regional carbon markets in China, and further demonstrates how forecast outputs can be translated into decision-support applications. The empirical results confirm that the proposed framework achieves strong predictive performance across most markets. Under the full-feature configuration, the weighted ensemble model attains an average RMSE of 2.638 CNY/t, MAE of 1.567 CNY/t, MAPE of 4.69%, and R^2^ of 0.908. Among the individual models, LightGBM delivers the highest average accuracy, while the ensemble approach ensures more stable and consistent performance across markets with heterogeneous characteristics.

A central insight of this study is that the effectiveness of external information varies significantly across markets. The feature ablation analysis reveals that no single predictor configuration dominates across all cases, highlighting the importance of market-specific information structures. Specifically, the full-feature model performs best in Shanghai and Hubei, while excluding macroeconomic variables improves performance in Beijing and Guangdong. Tianjin benefits from removing cross-market linkage features, Fujian from excluding trading volume, and Shenzhen from relying solely on price-based inputs. These findings suggest that carbon price dynamics in China are shaped not only by modeling techniques but also by differences in market structure and information relevance.

Beyond predictive accuracy, the framework extends carbon price forecasting into an application-oriented analytical paradigm. The forecasted price trajectories are utilized to identify favorable timing strategies for allowance selling and to evaluate dynamic carbon costs in offshore wind scour protection scenarios. This integration demonstrates how predictive models can support both market operations and engineering-related decision processes.

Despite these contributions, several limitations remain.

(1) The preprocessing procedure uses full-calendar linear interpolation for missing price values, which may introduce a limited look-ahead risk because future observations can be involved in the interpolation process. Although the proportion of missing price observations is low and the same preprocessing protocol is applied consistently across markets, the reported results should be interpreted as a controlled empirical comparison rather than as a fully leakage-free operational forecasting evaluation. Future work should adopt strictly time-causal imputation methods, such as forward filling within each training, validation, and test segment.
(2) The benchmark set in this study is limited to representative tree-based machine learning models and their weighted ensemble. Classical econometric and statistical forecasting benchmarks, such as ARIMA, GARCH, and random-walk-type models, are not fully examined. This limits the ability to quantify the incremental predictive gain of the proposed framework relative to traditional carbon-price forecasting approaches. Future work should incorporate these benchmarks into the same cross-market evaluation protocol.
(3) Some differences across feature-ablation configurations are numerically small. Since formal forecast-comparison tests, such as Diebold–Mariano tests, bootstrap confidence intervals, or rolling-window robustness checks, are not implemented, the ablation results should be interpreted as indicative evidence of market-dependent feature relevance rather than as statistically definitive proof of distinct information structures.
(4) Chinese ETS markets are strongly affected by regulatory announcements, compliance cycles, allowance-allocation rules, institutional interventions, and other policy shocks. These policy-related factors are not explicitly incorporated in the present feature set. As a result, the model may not fully capture abrupt price movements triggered by regulatory events. Future work could incorporate policy-event dummy variables, compliance-calendar indicators, allowance-allocation information, or text-based features from policy announcements.
(5) The multi-step forecasting strategy relies on recursive prediction, leading to error accumulation as the forecast horizon increases. While one-step-ahead forecasts show relatively strong accuracy within the tree-based benchmark considered in this study, the 30-day recursive predictions introduce greater uncertainty over time. Future work should quantify error propagation through multi-step RMSE analysis and determine effective forecasting horizons for different applications.
(6) Model hyperparameters are kept consistent across all markets to enable controlled comparison. Although this setting helps isolate cross-market and feature-group effects, market-specific tuning may further enhance performance, especially in structurally distinct markets such as Shenzhen and Guangdong. More complex hybrid ensemble models, metaheuristic optimization algorithms, and adaptive feature-selection strategies could also be incorporated in future work. However, these extensions should be evaluated carefully to avoid shifting the focus from market-dependent feature relevance to algorithmic benchmarking alone.
(7) The decision-support applications presented in this study are exploratory in nature and are not designed as fully optimized operational systems. In particular, the LCA-based offshore wind case is used to demonstrate how forecast carbon prices can be linked with life-cycle emission estimates, rather than to provide a complete standalone LCA assessment. Future research could further develop the LCA component as an independent study with more detailed engineering inventory data, site-specific uncertainty analysis, and broader engineering scenarios.

In summary, this study provides a systematic cross-market evaluation of carbon price forecasting in China within a controlled tree-based machine learning framework. The results suggest that the usefulness of external information varies across regional carbon markets, and that no single feature configuration consistently gives the lowest prediction error across all cases. The decision-support extension further illustrates how forecast price paths can be translated into allowance-selling and engineering carbon-cost signals when relevant decision parameters are available. However, these outputs should be interpreted as exploratory decision-support signals rather than fully validated operational recommendations. More broadly, this study suggests that carbon price forecasting in heterogeneous markets requires attention not only to model selection, but also to the suitability of different feature groups under market-specific conditions.

## Figures and Tables

**Table 1:** Overview of the seven regional carbon markets included in this study.

Market	Launch	Observations	Activity	Liquidity Characteristics
Shanghai	2013	2400	High	Highly active; continuous trading, dynamic prices
Beijing	2013	2400	High	Highly active; continuous trading, dynamic prices
Guangdong	2013	2400	High	Active trading; relatively dynamic price movements
Hubei	2014	2200	Medium	Relatively stable price formation
Tianjin	2013	2200	Low	Heterogeneous trading patterns; weak liquidity
Shenzhen	2013	2524	Low	Heterogeneous trading patterns; weak liquidity
Fujian	2016	1607	Low	Latest launch; smallest sample

**Table 2:** The 16 macroeconomic and energy variables used in the baseline framework.

Economic Activity (5)	Energy & Power (5)	Derived & Momentum (6)
Manufacturing PMI	Coal futures closing price	Coal futures return
Services PMI	Coal futures trading volume	Coal futures 20-day MA
PPI (YoY growth)	Power plant coal inventory	Coal price 5-day MA
CPI (YoY growth)	Daily coal consumption	Coal price momentum
Industrial value-added growth	Available power days	PMI deviation from 50
		PPI–CPI spread

**Table 3:** Feature group inclusion across the five ablation configurations.

Feature Group	M1 Full	M2 No-Vol	M3 No-CM	M4 No-Mac	M5 Price-Only
G1 Price & Technical	✓	✓	✓	✓	✓
G2 Trading Volume	✓	✗	✓	✓	✗
G3 Cross-Market	✓	✓	✗	✓	✗
G4 Macroeconomic (16 vars)	✓	✓	✓	✗	✗

Note: ✓ = included; ✗ = excluded. M1 is the full model and serves as the baseline.

**Table 4:** Hyperparameter settings of the three base learners.

Model	Parameter	Value
XGBoost	Learning rate	0.01
XGBoost	Maximum boosting rounds	1000
XGBoost	Early stopping patience	70
XGBoost	Maximum tree depth	4
XGBoost	L1 regularisation coefficient	0.1
XGBoost	L2 regularisation coefficient	1.0
LightGBM	Learning rate	0.01
LightGBM	Maximum boosting rounds	1000
LightGBM	Early stopping patience	70
LightGBM	Number of leaves	31
LightGBM	Minimum child sample size	8
Random Forest	Number of trees	300
Random Forest	Maximum depth	6
Random Forest	Minimum leaf size	4

Note: These hyperparameters are fixed across all seven regional carbon markets to ensure consistency in cross-market comparison.

**Table 5:** Training and evaluation settings of the forecasting framework.

Component	Setting	Value
Data split	Training: Validation: Test	75:12:13
Prediction target	Forecast variable	Next-day price change
Evaluation basis	Metrics computed on	Reconstructed price levels
Ensemble	Ensemble-weight optimization method	Validation-set grid search
Ensemble	Weight selection criterion	Validation-set MSE
Macro preprocessing	Smoothing method	7-day moving average
Multi-step forecasting	Updating strategy	Recursive updating
Evaluation metrics	Reported metrics	RMSE, MAE, MAPE, R^2^

Note: All evaluation metrics are computed on reconstructed price levels rather than on predicted price changes.

**Table 6:** (**A**) Scenario-level life-cycle carbon emissions of the two scour protection strategies (202 MW offshore wind farm, 25-year design life). (**B**) Full-sample descriptive statistics of daily allowance (CEA) prices and trading activity for Guangdong and Hubei.

(A)			
Strategy	Scenario	GWP (kg CO_2_-eq/MWh)	E (tCO_2_-eq, 25 yr)
S1 (Rock dumping)	Low (2 int./25 yr)	4.44	56,503
	Mid (5 int./25 yr) *	4.55	57,903
	High (8 int./25 yr)	4.71	59,939
S2 (Cement-stabilised soil)	Fast stabilisation	8.15	103,717
	Mid stabilization *	9.94	126,496
	Slow stabilisation	13.51	171,918
**(B)**			
**Statistic**	**Guangdong**	**Hubei**	**Interpretation**
Price mean/SD (CNY/t)	30.93/21.32	27.55/10.39	Wider dispersion in Guangdong
Price coefficient of variation	69.0%	37.7%	Guangdong ≈ 1.8 × Hubei
Price range (min–max, CNY/t)	8.10–95.26	10.48–61.89	Larger span in Guangdong
Daily log-return SD	7.73%	4.10%	Stronger volatility in Guangdong
Share of days with |return| > 5%	23.2%	13.7%	More large jumps in Guangdong
Return excess kurtosis	48.0	14.6	Heavy-tailed returns
Max single-day price change (CNY/t)	49.75	12.34	Larger jump in Guangdong
Avg. trading days per full year	≈243	≈243	Similar trading-day count
Trading-volume CV	245%	255%	Bursty trading in both markets
Volume share of busiest 5% days	45%	37%	Concentrated trading

Note: (A) * denotes the baseline scenario. GWP values are normalised to the functional unit (1 MWh net electricity, 25-year life). E values are total 25-year site-level emissions. (B) Statistics are calculated from full-sample valid CEA daily observations. CV = coefficient of variation, calculated as SD/mean. Daily volatility is measured as the standard deviation of daily log-returns. Excess kurtosis is reported relative to the normal distribution, for which excess kurtosis equals 0. Volume CV and the volume share of the busiest 5% of days are used to characterize the concentration and lumpiness of trading activity.

**Table 7:** Baseline model performance across seven regional carbon markets (full-feature M1 configuration).

Market	Metric	XGBoost	LightGBM	Random Forest	Ensemble
**Shanghai**	RMSE	1.2623	1.2615	1.4527	1.2658
	R^2^	0.9630	0.9630	0.9510	0.9628
	MAE	0.7012	0.6980	1.0068	0.7068
	MAPE	1.29%	1.29%	1.83%	1.30%
**Beijing**	RMSE	6.7398	6.7319	6.8578	6.7558
	R^2^	0.9440	0.9442	0.9420	0.9438
	MAE	3.7773	3.8701	4.3251	4.0330
	MAPE	4.40%	4.53%	5.01%	4.69%
**Guangdong**	RMSE	4.0847	4.1134	4.2151	4.1100
	R^2^	0.7504	0.7468	0.7342	0.7473
	MAE	1.8873	1.9228	2.0720	1.8923
	MAPE	2.78%	2.83%	2.99%	2.78%
**Hubei**	RMSE	1.3919	1.2404	1.7306	1.2743
	R^2^	0.7825	0.8273	0.6638	0.8177
	MAE	0.9989	0.7716	1.3649	0.8152
	MAPE	2.08%	1.61%	2.84%	1.70%
**Tianjin**	RMSE	0.6134	0.6162	0.6663	0.6132
	R^2^	0.9783	0.9781	0.9744	0.9784
	MAE	0.2444	0.2292	0.3698	0.2478
	MAPE	0.77%	0.72%	1.11%	0.77%
**Fujian**	RMSE	1.3304	1.3326	1.3799	1.3352
	R^2^	0.9261	0.9258	0.9205	0.9255
	MAE	1.0174	1.0202	1.0370	1.0215
	MAPE	3.99%	4.00%	4.05%	4.00%
**Shenzhen**	RMSE	3.0490	3.1694	3.2898	3.1084
	R^2^	0.9785	0.9767	0.9749	0.9776
	MAE	2.1825	2.2834	2.5146	2.2533
	MAPE	16.68%	17.53%	20.76%	17.61%
**Mean**	RMSE	2.6388	2.6379	2.7989	2.6375
	R^2^	0.9033	0.9088	0.8801	0.9076
	MAE	1.5156	1.5422	1.8129	1.5671
	MAPE	4.57%	4.64%	5.51%	4.69%

Note: RMSE and MAE are in CNY/t. Bold market names indicate the first row of each market block. Shenzhen MAPE is elevated due to an early low-price regime.

**Table 8:** (**A**) Ablation results (RMSE and R^2^) across five feature configurations and seven markets. (**B**) Summary of best-performing ablation configuration per market.

(A)						
Market	Metric	M1	M2	M3	M4	M5
Shanghai	R^2^	0.9628	0.9625	0.9616	0.9628	0.9626
	RMSE	1.2658	1.2707	1.2864	1.2661	1.2697
Beijing	R^2^	0.9438	0.9432	0.9435	0.9441	0.9439
	RMSE	6.7558	6.7873	6.7730	6.7340	6.7461
Guangdong	R^2^	0.7473	0.7503	0.7402	0.7534	0.7492
	RMSE	4.1100	4.0853	4.1668	4.0601	4.0942
Hubei	R^2^	0.8177	0.8077	0.7924	0.7799	0.7599
	RMSE	1.2743	1.3087	1.3599	1.4002	1.4625
Tianjin	R^2^	0.9784	0.9783	0.9784	0.9783	0.9783
	RMSE	0.6132	0.6145	0.6131	0.6142	0.6137
Fujian	R^2^	0.9255	0.9266	0.9255	0.9258	0.9260
	RMSE	1.3352	1.3259	1.3354	1.3331	1.3310
Shenzhen	R^2^	0.9776	0.9754	0.9811	0.9755	0.9816
	RMSE	3.1084	3.2581	2.8606	3.2539	2.8199
**(B)**						
**Market**	**Best-Config.**	**Best-RMSE**	**Interpretation**			
Shanghai	M1	1.2658	Full model marginally best; all feature groups contribute in a balanced way			
Beijing	M4	6.7340	Active policy market; macro already priced in			
Guangdong	M4	4.0601	Cross-market and volume features beneficial; macro adds noise			
Hubei	M1	1.2743	Full model best; all external information contributes positively			
Tianjin	M3	0.6131	Stable, illiquid; cross-market features add marginal noise			
Fujian	M2	1.3259	Low-continuity trading; volume adds noise			
Shenzhen	M5	2.8199	Distinct early low-price regime; cross-market linkage destabilising			

**Table 9:** Interpretation of engineering preference under the forecast carbon price path and threshold region.

Forecast-Threshold Relation	Interpretation	Engineering Implication
Clearly below $P^{*}$	Higher-emission option likely retains total-cost advantage	Direct-cost advantage dominates
Close to or overlapping $P^{*}$	Ranking becomes sensitive to forecasting and scenario assumptions	Conditional preference
Clearly above $P^{*}$	Lower-emission option may become preferable in total-cost terms	Carbon pricing reshapes design preference

Note: P* denotes the carbon price threshold at which the total-cost preference between the two engineering options may change.

## Data Availability

Data are contained within the article.
